# New Horizons in Antifungal Therapy

**DOI:** 10.3390/jof2040026

**Published:** 2016-10-02

**Authors:** Kaila M. Pianalto, J. Andrew Alspaugh

**Affiliations:** 1 Department of Molecular Genetics and Microbiology, Duke University School of Medicine, Durham, NC 27710, USA; kaila.pianalto@duke.edu; 2 Department of Medicine/Infectious Diseases, Duke University School of Medicine, Durham, NC 27710, USA

**Keywords:** amphotericin, polyene, azole, echinocandin, flucytosine, aspergillosis, candidiasis, cryptococcosis, fungal infection, mycosis

## Abstract

Recent investigations have yielded both profound insights into the mechanisms required by pathogenic fungi for virulence within the human host, as well as novel potential targets for antifungal therapeutics. Some of these studies have resulted in the identification of novel compounds that act against these pathways and also demonstrate potent antifungal activity. However, considerable effort is required to move from pre-clinical compound testing to true clinical trials, a necessary step toward ultimately bringing new drugs to market. The rising incidence of invasive fungal infections mandates continued efforts to identify new strategies for antifungal therapy. Moreover, these life-threatening infections often occur in our most vulnerable patient populations. In addition to finding completely novel antifungal compounds, there is also a renewed effort to redirect existing drugs for use as antifungal agents. Several recent screens have identified potent antifungal activity in compounds previously indicated for other uses in humans. Together, the combined efforts of academic investigators and the pharmaceutical industry is resulting in exciting new possibilities for the treatment of invasive fungal infections.

## 1. Introduction

Fungal infections are a worldwide global health problem, affecting millions of patients per year [1]. Of these, approximately 1.5 million are disseminated or invasive fungal infections (IFIs), requiring advanced treatment and hospitalization [1]. Unfortunately, this high number of infections is associated with high mortality rates, with some fungal infections having mortality rates nearing 90%–95% [2,3]. A summary of some of the most common fungal diseases along with their rates of incidence and mortality can be seen in Table 1. Worldwide, most IFIs are caused by the *Candida*, *Cryptococcus*, *Aspergillus* and *Pneumocystis* species, although diseases caused by rarer fungi are becoming more common. Additionally, the thermally-dimorphic, or endemic, fungi, which tend to be more prevalent in specific geographic zones, have high rates of unreported infection due to the frequency of subclinical infection [4,5].

Generally, IFIs are infections of immunocompromised hosts. The standard definition of the immunocompromised host is expanding from the traditional set of patients with AIDS, patients with cancer who are undergoing immunosuppressive chemotherapy or transplant patients whose immune systems are suppressed to prevent organ rejection [26]. IFIs can also be seen during treatment with new biologically-active agents, such as TNF-α inhibitors, used to treat autoimmune or inflammatory diseases. These inhibitors dampen inflammation and help treat disease symptoms, but they can also lead to opportunistic infections [27]. Additionally, IFIs are also observed in patients who are healthy and apparently immunocompetent, but who have underlying, asymptomatic conditions that might alter immune function and predispose toward infection, such as the presence of autoantibodies against cytokines, such as GM-CSF [28].

Due to the high global health burden associated with fungal disease, the treatment of these infections needs to be potent and effective. Indeed, many of the currently available classes of antifungal drugs are highly effective in the appropriate contexts. However, these drugs, as with any therapy, have limitations and caveats. For example, the toxicities associated with the use of some antifungal agents can be prohibitive toward use or must be accepted in order to effectively treat the patient. Additionally, there are few approved antifungal agents in only four drug classes for the treatment of IFIs. Further limiting is the small number of targets that these drugs act upon, owing to the high levels of similarity between the eukaryotic fungal pathogens and the human hosts. For example, of the four classes of antifungal drug approved for treatment of invasive fungal infections, two of these, polyenes and the azoles, target the same component of the fungal cell membrane [29,30]. This small number of cellular targets increases opportunities for fungi to develop resistance to one or more of the available antifungals. Furthermore, few of the currently available drugs are actually fungicidal. Finally, several of these drugs have limitations in geographic availability, particularly in areas with high rates of fungal infections [31]. This leads to higher mortality rates for IFIs due to treatment with less effective antifungal drugs.

Moreover, the problem of antifungal resistance is on the rise: both that which has evolved in formerly sensitive species, as well as the prevalence of intrinsically-resistant species of fungi. To date, resistance exists to all of the currently available classes of antifungal agent [32,33,34,35,36]. *Candida* species have a high prevalence of azole resistance, largely attributed to the cytostatic nature of these drugs [37,38]. Similarly, *Aspergillus* and *Cryptococcus* strains have recently also demonstrated azole resistance [35,39,40,41]. Only a few years ago, echinocandins were considered effective therapy for most clinically-relevant *Candida* isolates. However, with increased use of these antifungal agents, echinocandin resistance in *Candida* species has also become more prevalent [36]. Additionally, the intrinsically drug-resistant fungi, such as *Scedosporium* species, continue to cause a background of infections in highly immunosuppressed patients, especially those who are heavily treated with antifungals. These infections are often associated with poor patient outcomes [42,43]. Due to these limitations, there is an urgent need for new antifungal agents.

Research goals for novel antifungal agents have emphasized a few major points. First, potency is a key characteristic of a novel drug. New drugs must be able to effectively control fungal growth in the context of the patient, at compound levels that are readily achievable at infection sites. Additionally, ideal novel antifungal agents should possess little to no host toxicity. Selectivity is also crucial, as the differences between the fungal pathogen and the human host are evolutionarily much smaller than those between bacterial pathogens and humans. Ideally, novel agents would be broad spectrum and able to treat multiple species of fungi. However, many antifungal compounds that are in development have potent, but very specialized, activity.

## 2. Antifungal Agents Approved for Clinical Usage

Currently, there are four major classes of antifungal drugs that are indicated for the treatment of invasive fungal infections. When used as indicated, these drugs can be highly effective at treating IFIs, with significant beneficial effects on patient mortality. A short summary of these drugs and their primary indications and usages can be found in Table 2.

### 2.1. Polyenes: Amphotericin B and Its derivatives

Amphotericin B and its newer lipid formulations are polyene antifungals that target the fungal plasma membrane. Recent models posit that these drugs act as “sponges” that bind to and remove ergosterol from the plasma membrane, reducing membrane integrity [30,44]. Due to its mechanism of action, amphotericin B is broad spectrum and indicated for the treatment of severe infections caused by *Candida* species, *Cryptococcus* species, Zygomycetes and as an alternative therapy for aspergillosis [45]. Amphotericin B is also used to treat many life-threatening IFIs due to other filamentous molds, as well as the thermally-dimorphic fungi, such as *Histoplasma*, *Coccidioides* and *Blastomyces*. Amphotericin B is cytocidal for most fungi. As amphotericin B is not highly bioavailable when administered orally, only intravenous (IV) formulations are used clinically. However, amphotericin B can have severe side effects, such as nephrotoxicity due to off-target binding of host membranes, limiting its usage to patients with life-threatening infections [46]. Newer formulations of this drug, such as the lipid-associated and liposomal formulations, demonstrate more selective fungal targeting and less host toxicity [46].

### 2.2. Azoles and Triazoles

Antifungal agents in the azole class target the fungal plasma membrane through inhibition of the biosynthesis of ergosterol, a fungal plasma membrane component that is similar to cholesterol found in mammalian cell membranes. This occurs through the inhibition of the sterol 14α-demethylase (cytochrome P450 51 or CYP51), which catalyzes the final step in ergosterol biosynthesis [29]. The inhibition of this enzyme leads to defects in fungal plasma membrane integrity and cellular integrity. The most commonly-used azoles for treating IFIs can be functionally divided between agents with primary activity against yeast-like fungi (yeast-active azoles), and those with expanded activity against fungi that often grow as molds (mold-active azoles). 

Fluconazole is the most widely-used yeast-active azole, and it is often very effective for treating infections caused by *Cryptococcus* and *Candida* species. Importantly, fluconazole resistance can present a significant clinical issue in systemic candidiasis: some *Candida* species, such as *C. krusei*, are intrinsically resistant to this drug, and other *Candida* isolates are often susceptible to this drug at high concentrations. Therefore, precise species identification and targeted antifungal susceptibility testing for clinically-relevant isolates are very important components of the care of patients with *Candida* IFIs.

The mold-active azoles include itraconazole, voriconazole, posaconazole and isavuconazole. In addition to retaining activity against *Candida* and *Cryptococcus* yeasts, these agents also inhibit many filamentous fungi. Itraconazole was the first available azole with significant activity against molds, such as *Aspergillus fumigatus*. However, issues with bioavailability and toxicity limit its current use for IFIs. Two newer agents, voriconazole and posaconazole, are more widely used for these infections, especially in highly immunocompromised patients. Voriconazole has become the first-line antifungal drug for treatment of invasive aspergillosis due to *Aspergillus fumigatus*. Comparative trials indicate that voriconazole is superior to many other antifungal agents for this infection [47]. Posaconazole is indicated for the prevention of IFIs, especially in the setting of prolonged neutropenia after high dose cancer chemotherapy. The use of these drugs has likely greatly improved outcomes in patients with invasive mold infections. However, both drugs have the potential to interact with other medications due to their inhibition of hepatic cytochrome P-450-dependent metabolism. Moreover, many azoles can result in cardiac conduction changes, and the QT interval should be monitored during therapy [48].

Isavuconazole (Cresemba^®^, Astellas Pharmaceuticals, Tokyo, Japan) is the most recently approved triazole antifungal drug. It differs from other approved azoles in several clinically-relevant ways. First, it has expanded in vitro activity that includes the Mucorales molds (Zygomycetes), such as *Rhizopus*, *Mucor* and *Cunninghamella* species, and it may, therefore, be an effective component of the complex, medical-surgical treatment of mucormycosis [49]. Additionally, the intravenous (IV) formulation of isavuconazole lacks cyclodextrin, a solubilizing agent used with other triazoles that is associated with nephrotoxicity in patients with renal insufficiency. Additionally, unlike other azole drugs isavuconazole does not appear to exacerbate QT prolongation, and it may actually shorten the QT interval in some patients [50].

### 2.3. Echinocandins

The echinocandins represent the newest class of antifungals. Currently, three drugs from this class are approved for clinical usage: caspofungin, micafungin and anidulafungin. Echinocandins affect cell wall biosynthesis through the noncompetitive inhibition of β-1,3-glucan synthase [51,52]. This enzyme is involved in the biosynthesis of one of the most abundant fungal cell wall components. Therefore, treatment with echinocandins leads to defects in fungal cell integrity. These drugs are primarily used for the treatment of invasive candidiasis and as an alternative therapy for treatment of aspergillosis [53]. Echinocandins have low host toxicity and few drug interactions. However, they have no activity against *Cryptococcus* species, and they are decidedly poor agents for treatment of the endemic mycoses. Additionally, they are not orally bioavailable, likely due to their large molecular size, and so, are only available in IV formulations.

### 2.4. 5-Fluorocytosine

5-fluorocytosine (flucytosine) is a fluoridated pyrimidine analog, which inhibits DNA and RNA synthesis by incorporating into the growing nucleic acid chain, preventing further extension. This nucleic acid damage eventually leads to cellular defects in protein biosynthesis and cell division. This antifungal agent has been attributed with cytostatic effects and high rates of resistance developing during monotherapy. Therefore, flucytosine is rarely used as a single agent for the treatment of fungal infections. However, it has been shown in multiple clinical trials to be highly effective in combination with amphotericin B for the treatment of cryptococcal meningitis [54,55]. Indeed, amphotericin B plus flucytosine is the first-line treatment for *Cryptococcus* central nervous system (CNS) infections [56]. Flucytosine can also be used in combination with other antifungals to treat *Candida* infections, though this is a less common practice. Adverse effects for flucytosine include bone marrow toxicity, especially in the presence of renal impairment. However, one of the truly limiting factors of this drug is its limited availability in countries with the highest incidence of cryptococcosis [31]. This, unfortunately, limits the effectiveness of cryptococcal meningitis therapy in those regions of the world in which it is most prevalent, likely increasing rates of mortality in this disease.

## 3. Antifungal Agents in Development with Novel Modes of Action

**Figure 1:**
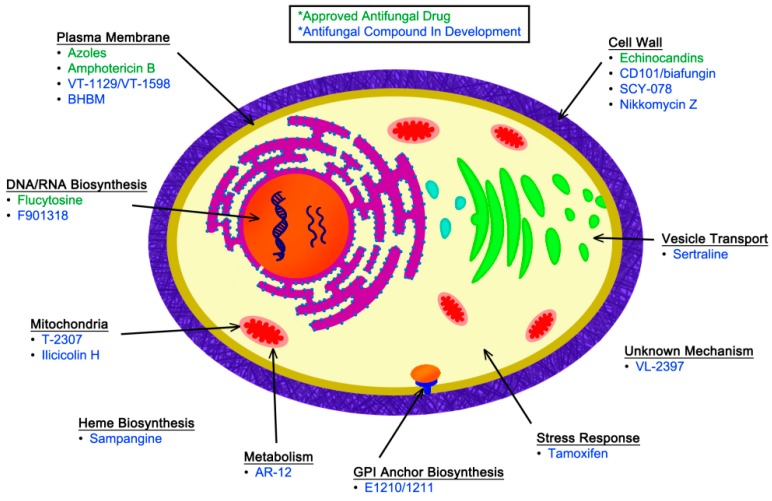
This diagram of a fungal cell indicates the sites of action for currently approved antifungal drugs (green) as well as other agents in various stages of development (blue).

In addition to the currently approved antifungal medications, novel compounds are in various stages of clinical development for the treatment of IFIs. These new agents have been identified in large-scale, unbiased screens for antifungal activity, as well as in targeted investigations based on detailed studies of fungal-specific cellular processes. In this review, we discuss selected novel antifungal compounds that have either begun to be assessed in clinical trials or that represent novel biological targets within fungi. These new compounds are discussed based on their known or predicted molecular target, illustrated in Figure 1. Although many investigators are also studying how to better harness the host immune response for effective treatment of IFIs, we will be focusing on therapeutics targeted against the fungal pathogen. A brief summary of the antifungal agents that will be discussed in this review, along with their activities based on minimum inhibitory concentration (MIC) and primary indications, can be found in Table 3.

### 3.1. Cell Membrane as Antifungal Target

#### 3.1.1. VT-1129, VT-1598 and VT-1161 (Viamet Pharmaceuticals)

VT-1129, VT-1598 and VT-1161 are compounds in a novel class of metalloenzyme inhibitors that, like the azoles and triazoles, inhibit the enzyme responsible for the final step of ergosterol biosynthesis: the fungal sterol 14α-demethylase (CYP51). These compounds were identified as part of an effort to decrease off-target binding of human CYP enzymes, including human CYP51. This has been achieved by identifying molecules, like the Viamet compounds, that are more specific for the fungal enzyme active site. Additionally, unlike the older generation azoles, whose high affinity for heme groups led to off-target inhibition of human CYP enzymes, the Viamet compounds have lower affinity for heme [83]. These features allow VT-1129 and VT-1161 to selectively inhibit fungal CYP51 over human CYP51. VT-1129 is approximately 3000-fold more selective for the *Cryptococcus* isoforms of CYP51 over human CYP51 in vitro, while VT-1161 is greater than 1000-fold more selective for the *Candida* enzyme. Theoretically, this increased fungal selectivity may decrease the risk for toxicity at higher doses of drug [81,84].

These compounds, like many of the azoles, are available in oral and intravenous forms. VT-1129 inhibits the growth of many Cryptococcus isolates, including both *C. neoformans* and *C. gattii* [80,81,85]. Additionally, in a mouse model of cryptococcal meningitis, treatment with VT-1129 led to dose-dependent clearance of *Cryptococcus* from the brain, performing better than fluconazole at similar drug concentrations [86]. Therefore, VT-1129 has been granted Qualified Infectious Disease Product (QIDP) designation, allowing expedited review for approval. VT-1129 is currently in phase 1 clinical trials for the treatment of cryptococcal meningitis.

VT-1598 is in preclinical development for treatment of coccidioidomycosis. Additionally, as part of Viamet’s extended platform, VT-1161, which has been shown to be effective against fluconazole-resistant *Candida* isolates, is in phase 2b clinical trials for the treatment of onychomycoses and recurrent vulvovaginal candidiasis [87].

#### 3.1.2. Inhibition of Membrane-Associated Lipids

*N*′-(3-bromo-4-hydroxybenzylidene)-2-methylbenzohydrazide (BHBM) and 3-bromo-*N*′-(3-bromo-4-hydroxybenzylidene) benzohydrazide (D0) represent a new class of antifungal compounds, termed “hydrazycins”. These agents were identified in a screen of synthetic compounds inhibiting sphingolipid biosynthesis in *C. neoformans*, a process demonstrated to be required for fungal growth in vivo [58]. These compounds inhibit vesicle trafficking of precursor lipids, such as ceramides, to the cell surface, thereby inhibiting glucosylceramide biosynthesis and cell division. Additionally, these compounds specifically inhibit fungal, but not human, glucosylceramide synthesis, suggesting fungal-specific cellular inhibition for this novel class of compounds. 

BHBM and D0 were identified in a screen for antifungal activity against *C. neoformans* at alkaline, but not acidic pH [88]. BHBM showed promising in vitro inhibitory activity against multiple isolates of two *Cryptococcus* species (*C. neoformans* and *C. gattii*), as well as *Histoplasma capsulatum*, *Blastomyces dermatitidis*, *Pneumocystis murinum* and *Pneumocystis jirovecii.* Notably, the strains tested included fluconazole-resistant *Cryptococcus* strains. In vivo activity for BHBM and D0 was assessed in murine models of infection due to *Cryptococcus neoformans*, *Candida albicans* and *Pneumocystis murinum*, resulting in significant increases in survival times compared to controls. Additionally, these drugs showed synergy with both fluconazole and amphotericin B, suggesting potential for combinatorial therapy. These hydrazycins were well tolerated in animal models of invasive fungal infection, though some interaction was observed with the immunosuppressive corticosteroid dexamethasone, which resulted in a decreased compound half-life in vivo [58].

### 3.2. Cell Wall Synthesis Inhibitors

#### 3.2.1. CD101 (Biafungin) (Cidara Therapeutics)

CD101, or biafungin, is a novel echinocandin formulated for both intravenous and topical use. It is similarly effective when compared to anidulafungin and caspofungin against *Aspergillus* and *Candida* species in vitro [59,60,61]. However, the advantage of CD101 over existing echinocandin drugs lies in its pharmacokinetics. The half-life of this drug is 81 h in vivo. In contrast, the half-life of anidulafungin, the longest-acting echinocandin to date, is approximately 24 h [89,90]. This allows biafungin to potentially be administered with once-weekly intravenous doses, rather than daily doses, better facilitating patient care and compliance while potentially decreasing drug administration costs. Biafungin, like other echinocandins, demonstrates few drug interactions and an excellent safety profile. This drug is currently in phase 2 clinical trials for the treatment of candidemia.

#### 3.2.2. SCY-078 (Scynexis)

SCY-078 is a novel β-1,3-glucan synthase inhibitor that is structurally distinct from the currently available echinocandin glucan synthase inhibitors. It is derived from enfumafungin, a novel natural product. Thereby, SCY-078 is a first-in-class, orally-available β-1,3-glucan synthase inhibitor that has received QIDP designation. An intravenous formulation is also in development. SCY-078 shows in vitro activity against isolates of *Candida* and *Aspergillus* species at MIC or MEC (Minimum Effective Concentration) levels below 0.5 µg/mL, including several fluconazole-resistant strains [70,72]. Additionally, SCY-078 remains active against certain echinocandin-resistant strains of *Candida* and *Aspergillus* [71,73]. When SCY-078 was tested in vitro against non-*Aspergillus* molds, SCY-078 was the only β-1,3-glucan synthase inhibitor with activity against the notoriously pan-resistant mold *Scedosporium prolificans* [91]. In a murine model of invasive candidiasis, treatment with SCY-078 led to a dose-dependent decrease in fungal burden in the kidneys across *Candida* species [72]. This drug is currently in phase 2 clinical trials in its oral formulation for the treatment of invasive candidiasis, while the IV formulation is in phase 1 clinical development. 

#### 3.2.3. Nikkomycin Z (University of Arizona)

Nikkomycin Z is an older drug that has resurfaced recently due to an increased interest in anti-cell wall antifungal drugs. Nikkomycin Z is a competitive inhibitor of chitin synthases, acting to decrease cell wall stability. Recently, it received Orphan Drug Status for its development as a treatment for coccidioidomycosis. In the past, this drug showed promise against the thermally-dimorphic fungi, including *Coccidioides immitis*, *Histoplasma capsulatum* and *Blastomyces dermatitidis* [68]. However, the clinical development of this drug was terminated due to difficulties in production. The drug was re-licensed to the University of Arizona, allowing for the reinstitution of clinical development. Nikkomycin Z is currently being tested in phase 1/2 clinical trials for treatment of coccidioidomycosis.

The renewed interest in Nikkomycin Z reflects momentum exploring the fungal cell wall as an antifungal target in general. This fungal-specific cellular feature makes for an ideal target for less toxic antifungal drug development.

### 3.3. Mitochondria as an Antifungal Target

#### 3.3.1. T-2307 (Toyama Chemicals, Tokyo, Japan)

T-2307 is a novel arylamidine compound that inhibits fungal growth by interfering with fungal metabolism. This compound specifically collapses fungal mitochondrial membrane potential, which prevents fungi from performing cellular respiration, thus compromising energy production for essential cellular processes [92]. Furthermore, this anti-mitochondrial activity is specific to fungi, and this drug does not collapse mammalian mitochondrial membrane potential at very high concentrations. The mechanism for this selectivity has been posited to be due to selective uptake of this drug by the high-affinity, fungal-specific Agp2 spermine/spermidine transporter [93]. T-2307, like many available antifungal drugs, has a fungistatic mechanism of action. However, it has potent in vitro activity against *Candida* species, *Cryptococcus neoformans*, *Aspergillus* species and *Fusarium solani*, including echinocandin-resistant *Candida* isolates [75,76,77]. The in vitro activity of T-2307 is comparable to the activity of voriconazole and micafungin for *Aspergillus* species and more effective at lower concentrations of drug than fluconazole, voriconazole and micafungin against *Candida*, *Cryptococcus* and *Fusarium* [75]. Additionally, in a murine model of systemic candidiasis, T-2307 performed as well as micafungin or amphotericin B, but at lower concentrations of compound [75].

#### 3.3.2. Ilicicolin H

Ilicicolin H acts on the mitochondria by specifically inhibiting the activity of the cytochrome bc1 complex. This inhibition of enzymatic activity decreases fungal mitochondrial respiration, preventing the biosynthesis of ATP. Based on in vitro data, fungal cytochrome bc1 is 50-fold more sensitive to ilicicolin H inhibition than the bovine enzyme and 1000-fold more sensitive than rat cytochrome bc1 [94,95]. Unfortunately, in some cases, resistance to this drug does occur [67]. Yet, this molecule is a promising starting point for future structural analogs with the potential for more specificity and potency against mitochondrial processes.

### 3.4. Other Mechanisms/Unknown Mechanisms

#### 3.4.1. VL-2397 (Vical, San Diego, CA, USA)

VL-2397 represents a new class of antifungal compound that has received QIDP designation for development for treatment of aspergillosis. Although the mechanism of fungal cell inhibition has yet to be determined, this compounds exhibits significant in vitro activity against *Aspergillus* species, *Cryptococcus neoformans*, *Candida glabrata*, *Candida kefyr* and *Trichosporon asahii*, as well as modest activity against *Fusarium solani* [79]. Additionally, VL-2397 shows fungicidal activity against *Aspergillus* species. However, this compound has no activity against other *Candida* species, including *C. albicans*, or any of the Mucorales fungi [79]. Interestingly, the import of the drug into fungal cells requires the siderophore transporter Sit1. As mammalian cells lack this transporter, this mechanism of entry could result in specificity for this drug toward fungal pathogens. 

VL-2397 has potent in vivo effects against azole-refractory *Aspergillus fumigatus* in a murine model of invasive aspergillosis [96]. Treatment with this drug resulted in a significant improvement in survival in infected mice compared with approved antifungal drugs, such as amphotericin B, posaconazole and caspofungin alone. Additionally, it is effective in combination with posaconazole in vivo, and posaconazole does not antagonize VL-2397 activity [96]. VL-2397 is currently in phase 1 clinical trials for the treatment of invasive aspergillosis. Although it demonstrates fairly narrow-spectrum activity, VL-2397 shows promise as a novel agent for combinatorial therapy, especially against *Aspergillus* species.

#### 3.4.2. AR-12 (Arno Therapeutics, Flemington, NJ, USA)

AR-12 was initially developed as an anticancer agent in 2006. This drug is a potent inhibitor of the phosphoinositide-dependent kinase PDK1 in humans and induces cell-death promoting endoplasmic reticulum stress, inhibiting proliferative cell growth [97]. However, AR-12 (OSU-03012) was identified through a repurposing screen of protein kinase inhibitors looking for agents that induced fungal cell lysis. This drug was thereby demonstrated to have potent antifungal activity [57]. It has since been granted the European Orphan Drug Designation for the treatment of cryptococcosis. Though a strong PDK1 inhibitor in humans, this drug does not inhibit the *C. neoformans* PDK1 [98]. However, based on haploinsufficiency profiling in *S. cerevisiae* and *C. albicans* (a microbial genetic technique used to test for potential mechanisms of antifungal action), it is suggested that AR-12 inhibits fungal carbon metabolism, specifically acetyl-CoA synthetase (ACS2) [98]. Inhibition of ACS2 leads to defects in several cellular processes, including histone acetylation, ribosome assembly and regulation of autophagy, in addition to its roles in carbon metabolism [98]. AR-12 shows activity against *C. albicans* biofilms, important structures in mucosal and catheter-associated infections. It is in preclinical development as an orally-available antifungal for the treatment of cryptococcosis.

#### 3.4.3. F901318 (F2G Ltd., Manchester, UK)

F901318 is a member of the novel orotomide class of antifungal drug that is formulated for both intravenous and oral delivery. It inhibits pyrimidine biosynthesis by blocking dihydroorotate dehydrogenase activity, preventing nucleotide biosynthesis. F901318 inhibits the growth of *Aspergillus* species in vitro, with MEC levels below 0.5 µg/mL [66]. It is even effective against azole- and amphotericin B-resistant *Aspergillus* strains [66]. In a murine model of invasive aspergillosis, F901318 treatment led to reduced galactomannan levels in mice, which has been shown to be associated with better clinical outcomes in patients [99,100]. F901318 is currently in phase 1 clinical trials assessing the safety of the IV formulation. Thus far, it has demonstrated an excellent safety profile and was well tolerated at therapeutically-relevant doses [101].

#### 3.4.4. E1210/1211

E1210 and E1211, which represent the active compound and its pro-drug, respectively, are inhibitors of fungal, but not human, glycophosphatidylinositol (GPI) anchor biosynthesis. This cellular process is required for the anchoring of proteins to both the fungal cell wall and cell membrane [64]. The anti-GPI activity of E1210 is achieved through inhibition of the fungal Gwt1 protein, which catalyzes an early step in the creation of the GPI anchor, while the human enzyme appears unaffected [64]. In *C. albicans*, treatment with E1210 inhibits germ tube formation, as well as biofilm formation and adherence to plastics [64]. E1210 has activity against *Candida*, *Aspergillus*, *Fusarium* and even *Scedosporium* species in vitro, in the ng/mL range for all strains tested [62,65]. E1210 also shows activity against azole- and echinocandin-resistant strains of *Candida* [102]. At 1–2 µg/mL, the activity appears to be fungicidal [63].

In a murine model of disseminated candidiasis, orally-administered E1210 had a significant, beneficial survival effect in animals infected with an azole-resistant strain of *C. albicans*. It also increased survival in mouse models of disseminated candidiasis and pulmonary aspergillosis, although this effect was seen at higher concentrations than that required for caspofungin, liposomal amphotericin B or fluconazole/voriconazole. E1210 also aided clearance versus an untreated control in a murine model of disseminated fusariosis [103].

#### 3.4.5. Sampangine

Sampangine is a copyrine alkaloid natural product which inhibits heme biosynthesis in vivo [69,104,105]. Because of this activity, treatment with this compound leads to defects in all heme-requiring pathways, including cellular respiration and ergosterol biosynthesis [69]. This natural product has very low MIC values for *Cryptococcus neoformans* (0.05 µg/mL), and it demonstrates inhibitory activity at concentrations of 3–6 µg/mL for *Candida* and *Aspergillus* species [69]. Synthesis of this and other alkaloids has been in development in the search for novel antifungals and antimicrobials, as specific heme biosynthesis inhibition could prove a potent target for antifungal development [106]. Indeed, some analogs that have been created have potent in vitro activity against *C. neoformans* and *A. fumigatus* [107].

### 3.5. Old Drugs, New Tricks

As demonstrated above, several avenues of research have identified novel compounds or classes of compounds that have significant antifungal effects, with the potential to develop toward novel therapeutics to be applied in a clinical setting. However, given the considerable resources required to develop completely novel antimicrobial drugs, many investigators have pursued a parallel strategy to identify existing medications that have unrecognized antifungal activity. This process of “redirecting” FDA-approved drugs for new antimicrobial indications holds promise to more quickly bolster our current, limited therapies for IFIs.

#### 3.5.1. Tamoxifen

Tamoxifen, an estrogen receptor agonist, has been used for decades in the treatment of estrogen receptor-positive breast cancer. However, in the early 1990s, it was discovered that tamoxifen has antifungal activity against *S. cerevisiae* and *C. albicans* [108,109]. More recently, in a screen of FDA-approved drugs, tamoxifen and its analog clomiphene were identified to be fungilytic toward *C. neoformans* [78]. Additionally, structural analogs of these triphenylethylene drugs were found to also have potent activity against several *Candida* and *Cryptococcus* species [78,110,111]. Using a yeast genetics system to identify the underlying mechanism behind the antifungal activity of these drugs, the target was determined to be the calcium-responsive signaling protein calmodulin. In fact, more potent fungal calmodulin inhibition correlated with more effective inhibition of *C. neoformans* growth [78,110]. Looking forward, modification and optimization of the triphenylethylene drugs may prove a promising avenue for antifungal therapy.

#### 3.5.2. Sertraline

Sertraline is an FDA-approved, selective serotonin reuptake inhibitor. The antimicrobial activity of sertraline was suggested when use of this antidepressant medication was associated with a decreased incidence of vulvovaginal candidiasis [112]. Further tests against *Candida* species revealed fungicidal activity in vitro, with minimum fungicidal concentrations of less than 29 µg/mL [112]. Minimum fungicidal concentrations (MFC_90_) for *Aspergillus* species were much higher (greater than 100 µg/mL) [113]. In 2011, sertraline was identified again in a screen for molecules potentiating fluconazole activity against a variety of yeasts [114]. 

Further studies of the antifungal activity of sertraline were conducted in *Cryptococcus* isolates, in which the MIC_90_ was 6 µg/mL or less, with of minimum fungicidal concentration of less than or equal to 10 µg/mL. In these studies, sertraline showed strain-dependent additivity or synergy with fluconazole [74]. Sertraline was found to decrease the cryptococcal burden in the brain and kidney compared to untreated or fluconazole-treated mice [74]. Consistent with previous studies, sertraline was found to be less potent when treating *Candida* species than *Cryptococcus* species, and it had antagonistic drug interactions with fluconazole in vitro [74]. Mechanistic genetic screening identified membrane trafficking and protein translation as potential targets of sertraline antifungal activity [74,114,115]. A clinical study in 2014 revealed faster clearance of fungal burden in the cerebrospinal fluid of patients with HIV-associated cryptococcal meningitis who were treated with sertraline in addition to standard antifungal therapy [116]. Sertraline is currently in phase 3 clinical trials as an adjunctive agent for the treatment of HIV-associated cryptococcal meningitis.

#### 3.5.3. Amphotericin B: New Compounds and Formulations

Amphotericin B is a highly effective fungicidal compound. However, it has had its history of significant toxicity in patients. As mentioned above, liposomal formulations of amphotericin B have decreased toxicity associated with treatment, but the potential for side effects remains even with these new formulations. However, new initiatives have focused on enhancing the safety of this important, broad-spectrum antifungal while maintaining efficacy. For example, REVOLUTION Medicines (Redwood City, CA, USA) has developed a platform, REVBLOCKS, by which it can rapidly modify and optimize natural products, such as amphotericin B, making novel synthetic compounds [117,118]. By functional group analysis, the Burke group (REVOLUTION) worked to understand the mechanisms by which amphotericin B kills yeast cells, as well as human cells. They identified functional groups that specifically bound fungal ergosterol, those that bound human cholesterol and those required for membrane pore formation. In this manner, it was discovered that ergosterol binding, but not pore formation, is the key fungicidal feature of amphotericin B [30,119,120]. This knowledge and their unique experimental platform enabled the design and synthesis of new amphotericin B analogs that specifically bind fungal ergosterol, but lack the pore-forming ability of the starting compound [30,120]. Certain urea-functionalized analogs of amphotericin B showed promise in a mouse model of candidemia, reducing fungal burden and mortality [118]. Preclinical studies are ongoing to identify promising candidate analogs of amphotericin B to move into further antifungal drug development.

In addition to these structural modifications, researchers have also been working on alternative forms of delivery for amphotericin B in order to aid in targeting of the drug to fungal pathogens, and not humans, as well as facilitating administration of this drug. For example, METAmphizon (nanomerics) is a nanoparticle formulation of amphotericin B that is currently in development. This drug formulation uses nanomerics’ Molecular Envelope Technology to encase cargo, such as a drug, in a self-assembling amphiphilic polymer. In the case of METAmphizon, the nanoparticle allows better organ targeting than amphotericin B to sites such as the lung, liver and spleen where fungal infections might reside [121]. Additionally, METAmphizon is available orally, unlike traditional amphotericin B formulations, and oral administration is similarly effective to both IV liposomal amphotericin B and IV METAmphizon in murine models of aspergillosis and candidiasis [121]. 

Other research has been looking at amphotericin B conjugates that take advantage of the “sterol sponge” activity of amphotericin B, while minimizing host toxicity from this molecule [30,44,122]. By conjugating amphotericin B with a “molecular umbrella”. It was possible to prevent amphotericin B from forming non-specific, membrane-disrupting aggregates at active concentrations while maintaining this drug’s ability to bind ergosterol [122]. The molecular-umbrella-amphotericin B conjugate displayed antifungal activity approaching that of the native compound. Importantly, the ability of the conjugate to lyse red blood cells and kidney cell lines in vitro was nearly abolished, results that show promise for in vivo experiments [122].

#### 3.5.4. Polymyxin B

Polymyxin B is a cationic lipid oligopeptide antibiotic that was identified in a screen of approved drugs for activity against *Aspergillus nidulans* [123]. However, upon further investigation, it was found to have relatively little effect on the fungal pathogens tested except for *Cryptococcus neoformans*, upon which it had a potent fungicidal effect and showed synergistic activity with fluconazole. Further study showed a potential mechanism for this species specificity, suggesting that the characteristic *Cryptococcus* polysaccharide capsule, an important virulence factor, is the target for the activity of polymyxin B [124]. In a mouse model of pulmonary cryptococcosis, polymyxin B reduces lung fungal burden alone, and more so in combination with fluconazole [124]. There has also been some investigation of the activity of other cationic peptide antibiotics against other fungal species [125,126,127,128].

## 4. Encouraging Targets from the Fungal Field

As our understanding of fungal pathogenesis has increased, the importance of certain virulence factors has become more apparent. The increase in knowledge about fungal virulence has led to further inquiry into how these virulence factors or processes could be leveraged as targets for antifungal drug design. In this case, the keys to antifungal drug design are to either identify processes that are specific to the fungal pathogen and that do not exist in the human host or to identify processes that may be conserved, yet have fungal-specific characteristics that can be manipulated for therapy. Though many potential “druggable” fungal targets have been identified, we will outline only a few in this article.

The idea of fungal-specific targets has been highly attractive, due to the low potential for off-target effects in the human host. Such has been the case with the cell wall-inhibiting echinocandins. Recent research has explored the trehalose biosynthesis pathway as a fungal-specific process required for virulence. Previous studies have shown that trehalose production is required for the virulence of several fungal species. For example, disruption of both *Candida albicans TPS1* and *TPS2*, the genes encoding trehalose-6-phosphate synthase and trehalose-6-phosphate phosphatase, respectively, leads to decreased growth at high temperatures, as well as a hyphal formation defect [129,130]. In *Aspergillus fumigatus*, the homolog to *TPS2*, the second gene in the trehalose biosynthetic pathway, is required for virulence, but has no impact on trehalose biosynthesis [131]. Other investigators determined that trehalose biosynthetic genes are required for high temperature growth and stress protection in both *Cryptococcus neoformans* and *C. gattii* [132,133]. Furthermore, deletion of the second gene involved in trehalose biosynthesis, *TPS2*, led to the accumulation of trehalose-6-phosphate and cell death, suggesting that blockade of biosynthesis at this point could provide a potent effect for treatment due to intermediate metabolite toxicity for the pathogen [133]. The recent determination of a crystal structure for *C. albicans* Tps2 allows for deeper understanding and modeling of compound-enzyme interactions, allowing for the identification of potential inhibitors that specifically bind to this unique target for antifungal drug development [134].

Additionally, there has been much interest in leveraging important, conserved signaling molecules for antifungal drug development by exploiting fungal-specific aspects of the components of these pathways. As an example, research on the Ras GTPases, though highly conserved among eukaryotes, has proven a promising avenue for antifungal drug development. In fungi, Ras GTPases play major roles in virulence. For example, in *C. neoformans*, Ras proteins are required for growth at high temperatures, a feature necessary for proliferation in the human host [135]. Additionally, in humans, mutations in Ras proteins are associated with many malignancies. Therefore, several inhibitors of Ras function are being actively explored as treatments for various cancers. These agents could serve as starting points to identify potent antifungal agents. For example, prenylation is a post-translational modification that is required for proper Ras protein localization and function in both mammals and fungi. Farnesylation is required for attachment of the RAS protein to cellular membranes and proper cellular localization [136,137,138]. Farnesyltransferase inhibitors (FTIs) have arisen somewhat recently as a promising class of anticancer drugs that would inhibit Ras activity due to protein mislocalization [139]. It has been proposed that some of these FTIs that have activity against cancer cell lines might also have antifungal activity [140]. As crystal structures are now available for both the mammalian and fungal farnesyltransferases, it is possible to apply this knowledge about these proteins toward developing therapeutics with specificity for either the human or the fungal enzyme [140,141,142]. These studies have shed light on structural and mechanistic differences in how the fungal and mammalian farnesyltransferases work, specifically during substrate binding, that can be leveraged toward the specific design of antifungal agents.

Other conserved eukaryotic pathways and proteins are being pursued as potential antifungal drug targets. Calcium/calmodulin signaling has shown promise as a target for antifungal therapy. In fungi, calcium/calmodulin signaling is an important regulator of stress responses, such as the response to high temperature, as well as, in some cases, resistance to antifungal drug treatment [143,144,145,146,147]. Additionally, calcineurin inhibitors, such as tacrolimus (Prograf/FK506), are already in use as immunosuppressants and have potent antifungal activity [148,149,150]. However, immunosuppression in the context of fungal infection is less than ideal. Development of non-immunosuppressive calcineurin inhibitors is an exciting avenue for antifungal agents.

Additionally, another conserved protein being explored as an antifungal drug target is the Hsp90 heat shock protein. This Hsp90 molecular chaperone is involved in protein folding in response to many cellular stresses. Fungal Hsp90 is notably involved in resistance to antifungals, including both azoles and echinocandins, in *Candida albicans*, *C. glabrata* and *Aspergillus fumigatus* [151]. Hsp90 inhibitors exert potent activity in combination with other antifungals. For example, the Hsp90 inhibitor geldanamycin has a potent fungicidal effect against azole-resistant *A. fumigatus* when used in combination with caspofungin or the calcineurin inhibitor FK506 [152]. Hsp90 inhibitors also potentiate the activity of the echinocandin micafungin, and they are fungicidal in combination with fluconazole against *C. albicans* in vitro and in an invertebrate model of candidiasis [153,154].

## 5. Promising Molecular Approaches to Antifungal Drug Discovery: Moving beyond Screening of Natural Products

In recent years, as seen above, there has been a push toward repurposing off-patent or FDA-approved drugs as antifungal agents. Researchers have also been working toward the identification of compounds that potentiate currently approved antifungal agents. Additionally, the concept of applying chemical genomics and large, high-throughput screening toward the goal of antifungal drug discovery has opened up promising avenues of study.

Multiple groups have been using small molecule libraries to screen for antifungal activity in a high-throughput manner. The Krysan group has developed an in vitro assay for rapidly assessing loss of cellular integrity, which measures the extracellular activity of the cytoplasmic enzyme, adenylate kinase, as a simple marker of cell lysis and fungal cell killing [155]. This assay has been used in multiple contexts to identify novel agents that disrupt cellular integrity in *C. neoformans*, some of which have been discussed above [78,156,157]. Other groups have used the alamarBlue^®^ assay in a similar manner to identify drugs that are fungicidal against *C. neoformans* [158] or to search for compounds that affected the viability of *Candida* biofilms [159].

Through a different approach, the Wright laboratory performed a screen for the potentiation of fluconazole activity against *C. neoformans*, *C. gattii*, *C. albicans* and *S. cerevisiae* [114], identifying several FDA-approved compounds that have synergy with fluconazole and potent activity against the fungi tested. More recently, this group has developed an Antifungal Combination Matrix, which arose from a screen of 3600 small molecules tested in combination with six approved antifungal compounds against four species of fungi: a dataset consisting of nearly 230,000 data points and around 86,000 chemical interactions [160]. This massive dataset can be leveraged toward identifying new agents that can increase the potency of existing antifungal agents. 

The Madhani group took a chemical genomics approach to a similar problem, using a large gene deletion library in *C. neoformans* to identify gene-drug interactions that could be leveraged toward antifungal therapy [161]. They identified gene-drug interactions for over 80% of the nearly 1500 gene deletion strains tested. They also defined the O2M algorithm, a decision-guiding process to analyze this gene-drug interaction data, in order to identify drugs that might act synergistically against *C. neoformans*. They used this “chemogenomic” profiling technique in comparison with *S. cerevisiae* datasets to identify conserved fungal responses. Interestingly, they found minimal conservation between the *C. neoformans* and *S. cerevisiae* datasets.

These studies bring an important point to light: there seems to be limited interspecies overlap of potentiators of fluconazole or other antifungals, nor was a significant amount of overlap found between *S. cerevisiae* and *C. neoformans* chemical genomics data. These data suggest that focusing on antifungal development that is geared toward particular pathogens, rather than focusing only on broad-spectrum activity, may lead to more potent and effective therapies for IFIs.

## 6. Conclusions

Invasive fungal infections represent a pressing global health problem. Although effective therapies exist for treatment of these diseases, resistance is common, and the mortality rates for IFIs are still unacceptably high. However, promising advances are being made in antifungal drug development, both through the development of novel compounds with potent antifungal activity and through the repurposing of previously described compounds for new uses as antifungal agents. Moreover, our expanding insight into the cellular processes required for fungal survival is now being translated to the specific identification of new therapeutic targets. Together, these efforts will greatly expand the currently limited number of drugs that we have to treat patients with life-threatening fungal infections.

## Figures and Tables

**Table 1:** Estimated yearly incidences of invasive fungal infections.

Fungal Disease	Estimated Cases per Year	Estimated Mortality Rates (% of Infected) ^1^
Cryptococcosis	>1,000,000 [6]	20%–70% [6]
Candidiasis	>400,000 [7]	10%–75% [8]
Aspergillosis	>200,000 [9,10]	30%–95% [1,10,11,12]
Pneumocystis Pneumonia	>400,000 [1,13]	20%–80% [13,14,15]
Mucormycosis (zygomycosis)	>11,000 [16]	30%–90% [16,17]
*Endemic/Dimorphic Fungi* ^2^		
Blastomycosis	~3000 [4]	<2%–68% [1,18,19]
Coccidioidomycosis	~20,000 [20]	<1%–70% [21]
Histoplasmosis	~25,000 [22]	28%–50% [5]
Paracoccidioidomycosis	~4000 [23]	5%–27% [23]
Penicilliosis	>8000 [1]	2%–75% [24,25]

^1^ Estimated mortality rates will vary widely depending on the immune competence of the host and geographical region (adapted from [1], with additional citations listed in the table); ^2^ estimated cases in endemic regions. Blastomycosis and histoplasmosis are endemic in the Midwestern U.S. and the Mississippi and Ohio River Valleys, coccidioidomycosis in the Southwestern U.S., paracoccidioidomycosis in Brazil and penicilliosis in Southeast Asia.

**Table 2:** Approved antifungal drugs for the treatment of invasive fungal infections.

Drug	Indication
*Polyenes*	
Amphotericin B	Life-threatening fungal infections, including cryptococcal meningitis, aspergillosis, blastomycosis and mucormycosis
*Azoles*	
Fluconazole	Invasive infections due to susceptible *Candida* species; cryptococcosis
Itraconazole	Blastomycosis, histoplasmosis, aspergillosis in patients refractory to Amphotericin B
Voriconazole	Invasive aspergillosis; non-neutropenic candidiasis; serious *Scedosporium* or *Fusarium* infections refractory to other agents
Posaconazole	Prevention of invasive fungal infections in neutropenic or HSC ^1^ transplant recipients
Isavuconazole	Invasive yeast and mold infections, including aspergillosis and mucormycosis
*Echinocandins*	
Caspofungin	Candidemia; refractory aspergillosis
Micafungin	Candidiasis
Anidulafungin	Candidiasis (adjunctive therapy with voriconazole for aspergillosis)
*Anti-metabolites*	
Flucytosine	Adjunctive therapy in *Cryptococcus neoformans* meningitis and *Candida* septicemia and endocarditis (in combination with amphotericin B)

^1^ HSC = hematopoietic stem cell.

**Table 3:** New antifungal agents in development.

Antifungal Compound	Indication(s) ^1^	Activity (MIC)	References
AR-12	*Cryptococcus neoformans*	4 µg/mL	[57]
	*Candida albicans*	4 µg/mL	
BHBM	*Cryptococcus neoformans*	0.25–8 µg/mL	[58]
	*Cryptococcus gattii*	0.5–2 µg/mL	
	*Candida glabrata*	0.125– >32 µg/mL	
	*Blastomyces dermatitidis*	0.5–1 µg/mL	
	*Histoplasma capsulatum*	0.125–1 µg/mL	
	*Pneumocystis jirovecii*	0.072–0.912 µg/mL ^2^	
CD101	Candidemia ^3,^*	≤0.008–2 µg/mL ^4^	[59,60,61]
	*Aspergillus* species ^5^	≤0.008–0.03 µg/mL ^4^	
E1210/1211	*Aspergillus* species ^5^	≤0.008–0.25 µg/mL	[62,63,64,65]
	*Candida* species ^6^	≤0.002–0.25 µg/mL	
	*Scedosporium* species	0.03–0.25 µg/mL	
	*Fusarium* species	0.015–0.25 µg/mL	
F901318	*Aspergillus* species ^5^	<0.03 µg/mL	[66]
Ilicicolin H	*Candida* species ^7^	0.01–5 µg/mL	[67]
	*Aspergillus fumigatus*	0.08 µg/mL	
	*Cryptococcus neoformans*	0.2–1.56 µg/mL	
Nikkomycin Z	Coccidioidomycosis *	0.125 µg/mL	[68]
Sampangine	*Cryptococcus neoformans*	<0.05 µg/mL	[69]
	*Candida albicans*	3.1 µg/mL	
	*Candida glabrata*	3.1 µg/mL	
	*Candida krusei*	6.2 µg/mL	
	*Aspergillus fumigatus*	6.2 µg/mL	
SCY-078	Invasive candidiasis *	0.03–2 µg/mL ^8^	[70,71,72,73]
	*Aspergillus* species ^5^	0.03–0.25 µg/mL ^4^	
Sertraline	*Cryptococcus* species *	2–6 µg/mL ^4^	[74]
T-2307	*Candida* species ^9^	0.00025–0.0078 µg/mL	[75,76,77]
	*Cryptococcus neoformans*	0.0039–0.0625 µg/mL	
	*Aspergillus* species ^5,10^	0.0156–2 µg/mL ^4^	
	*Fusarium solani*	0.125 µg/mL	
	*Mucor racemosus*	2 µg/mL	
Tamoxifen	*Cryptococcus neoformans*	64 µg/mL	[78]
	*Candida albicans*	32 µg/mL	
	*Candida glabrata*	8 µg/mL	
VL-2397	Invasive aspergillosis *	1–4 µg/mL ^4,9^	[79]
	*Candida glabrata*	≤2 µg/mL	
	*Candida kefyr*	≤2 µg/mL	
	*Cryptococcus neoformans*	≤2 µg/mL	
VT-1129	Cryptococcal meningitis *	<0.0001–0.25 µg/mL ^4^	[80,81,82]
	*Candida* species ^11^	<0.0001–1 µg/mL	
VT-1598	Coccidioidomycosis	NA ^12^	NA

^1^ Indication for agents in clinical trials, denoted by *. For preclinical agents, organisms for which there is significant antifungal activity are listed. ^2^ IC_50_ = 50% inhibition. MIC not determined due to culture conditions. ^3^ Includes *Candida albicans*, *C. glabrata*, *C. tropicalis*, *C. krusei*, *C. parapsilosis*, *C. dubliniensis* and *C. orthopsilosis* isolates. ^4^ Represents MIC_90_, or MIC for ≥90% of isolates tested, with occasional resistant isolates observed. ^5^ Includes *Aspergillus fumigatus*, *A. terreus*, *A. flavus* and *A. niger* isolates. ^6^ Includes *Candida albicans*, *C. glabrata*, *C. tropicalis*, *C. parapsilosis* and *C. dubliniensis* isolates. ^7^ Includes *Candida albicans*, *C. glabrata*, *C. guillermondii*, *C. krusei*, *C. lusitaniae* and *C. parapsilosis* isolates. ^8^ Includes *Candida albicans*, *C. glabrata*, *C. tropicalis*, *C. parapsilosis* and *C. krusei* isolates. ^9^ Includes *Candida albicans*, *C. dubliniensis*, *C. glabrata*, *C. guillermondii*, *C. krusei*, *C. parapsilosis* and *C. tropicalis* isolates. ^10^ Includes *Aspergillus fumigatus*, *A. flavus*, *A. terreus* and *A. nidulans* isolates. ^11^ Includes *Candida albicans*, *C. glabrata* and *C. krusei* isolates. ^12^ NA: MIC information not publicly available.

## Abbreviations

The following abbreviations are used in this manuscript:

IFI	Invasive fungal infection
IV	Intravenous
CYP	Cytochrome P450
CNS	Central nervous system
MIC	Minimum inhibitory concentration
QIDP	Qualified Infectious Disease Product
BHBM	*N*′-(3-bromo-4-hydroxybenzylidene)-2-methylbenzohydrazide
D0	3-bromo-*N*′-(3-bromo-4-hydroxybenzylidene) benzohydrazide
GPI	Glycophosphatidylinositol
MEC	Minimal effective concentration
MFC	Minimal fungicidal concentration
FTI	Farnesyltransferase inhibitor
